# Evaluation of Wood Decay and Identification of Fungi Found in the USS *Cairo*, a Historic American Civil War Ironclad Gunboat

**DOI:** 10.3390/jof11100732

**Published:** 2025-10-11

**Authors:** Robert A. Blanchette, Benjamin W. Held, Claudia Chemello, Paul Mardikian

**Affiliations:** 1 Department of Plant Pathology, University of Minnesota, St. Paul, MN 55108, USA; bheld@umn.edu; 2 Terra Mare Conservation LLC, Charleston, SC 29403, USA; claudia@terramareconservation.com (C.C.); paul@terramareconservation.com (P.M.)

**Keywords:** Ascomycota, Basidiomycota, biodegradation, conservation of cultural heritage, fungi, historic shipwreck, microbial ecology

## Abstract

Studies of microbial degradation of historic woods are essential to help protect and preserve these important cultural properties. The USS *Cairo* is a historic Civil War gunboat and one of the first steam-powered and ironclad ships used in the American Civil War. Built in 1861, the ship sank in the Yazoo River of Mississippi in 1862 after a mine detonated and tore a hole in the port bow. The ship remained on the river bottom and was gradually buried with sediments for over 98 years. After recovery of the ship, it remained exposed to the environment before the first roofed structure was completed in 1980, and it has been displayed under a tensile fabric canopy with open sides at the Vicksburg National Military Park in Vicksburg, Mississippi. Concerns over the long-term preservation of the ship initiated this investigation to document the current condition of the wooden timbers, identify the fungi that may be present, and determine the elemental composition resulting from past wood-preservative treatments. Micromorphological characteristics observed using scanning electron microscopy showed that many of the timbers were in advanced stages of degradation. Eroded secondary cell walls leaving a weak framework of middle lamella were commonly observed. Soft rot attack was prevalent, and evidence of white and brown rot degradation was found in some wood. DNA extraction and sequencing of the ITS region led to the identification of a large group of diverse fungi that were isolated from ship timbers. Soft rot fungi, including *Alternaria*, *Chaetomium*, *Cladosporium*, *Curvularia*, *Xylaria* and others, and white rot fungi, including *Bjerkandera*, *Odontoefibula*, *Phanerodontia*, *Phlebiopsis*, *Trametes* and others, were found. No brown rot fungi were isolated. Elemental analyses using induced coupled plasma spectroscopy revealed elevated levels of all elements as compared to sound modern types of wood. High concentrations of boron, copper, iron, lead, zinc and other elements were found, and viable fungi were isolated from this wood. Biodegradation issues are discussed to help long-term conservation efforts to preserve the historic ship for future generations.

## 1. Introduction

Microbial degradation of wood may involve fungi causing different types of decay as well as degradation by bacteria depending on environmental conditions [1,2,3]. Any wood placed in an outdoor environment, even under extreme environmental conditions, will be affected by microbial decay [4,5,6,7]. Although decomposition is an essential part of the natural recycling of organic substances within the Earth’s ecosystems, microbial degrading agents can be detrimental to wood that is used in buildings, ships or other wooden structures [8,9]. The preservation of historic wood is of particular concern since it can have great cultural significance and can be subjected to many different degradative processes over long time periods [10]. A great deal of information has become available on the mechanisms of wood decay in forests and wood products, but more knowledge is needed to better understand microbial degradation that affects historic wood. To control decay in historic wood so that successful conservation and preservation can be realized, it is important to obtain as much information as possible about the microbes present and what impact they have had on the wood. This paper focuses on the USS *Cairo*, a historic Civil War gunboat that is currently a national treasure and tourist attraction at the Vicksburg National Military Park in Mississippi.

The American Civil War was a time of naval advancements, with the production of the first steam-powered wooden ships with iron armor. The USS *Cairo* was one of the Union City Class Mississippi squadron gunboats built in 1861 [11]. Its design was tailored so it could navigate shallow rivers, allowing for combat on the Mississippi and Ohio Rivers as well as their tributaries. During a mine clearing operation in 1862, the gunboat struck a mine, which tore a hole in the bow, sinking the ship [12]. Over the years, silt, sand and mud completely covered the ship. It was not until over 98 years later that recovery efforts began, and the historic ship was raised from the bottom of the Yazoo River in Mississippi [11].

It is now well known that wood degradation takes place in all environments, including waterlogged wood buried under sediment [13,14]. A combination of soft rot attack by fungi as well as erosion and tunneling degradation by bacteria can occur in wood from sunken ships [13,14,15,16]. Soft rot attack, characterized by cavity formation within the secondary cell wall layers of wood cells [1,17], occurs in exposed wood that is on the river bottom and in wood that is not covered deeply with sediment [14]. As sediments accumulate, however, and the wood becomes buried, conditions change, with less oxygen present. Soft rot attack then becomes limited, and bacterial degradation becomes more prevalent [2,14]. Assuming that the ship was covered by a progressive buildup of sediments over time, a combination of soft rot fungal attack and bacterial degradation can be assumed.

When wood remains waterlogged, the effects of degradation may not be visually evident but significant strength losses to the wood can occur [16]. During recovery efforts in the 1950s, divers reported that the wood of the USS *Cairo* appeared to be in good condition [11]. However, when cables and wires were used to try and lift the ship, the hull did not possess sufficient strength to resist the cutting force from the lifting wires, and the ship was cut through in several places. It was reported that the ship wood was cut as if one were to cut through cheese with a wire [11]. This strongly suggests that the wood had become substantially degraded while being buried in sediment on the river bottom.

Funding for preservation and restoration was not available for over 13 years after recovery. During this time, limited conservation efforts took place and additional degradation of the ship occurred. A summary of the restoration efforts has been previously reported [11,18,19]. For over a decade, the ship was being stored unsheltered and exposed to rain and outside environmental conditions in Mississippi. The ship was also intermittently sprayed with freshwater, and it was reported that this produced a microenvironment conducive to rot [11]. These conditions would likely support decay by white and brown rot fungi [10]. When funding was secured for preserving the ship at the Vicksburg National Military Park under the direction of the US National Park Service, it was placed under a covered structure and the wood received various treatments, including pentachlorophenol and boron preservative, to protect it from insects and decay [11].

The ship has remained at Vicksburg for over 40 years, and new conservation efforts have been initiated to better understand the current condition of the ship’s wood and to determine what fungi are present that may be detrimental. The objectives of this study were to determine the elemental composition and micromorphological condition of the wood and to identify the fungi that were cultured using DNA sequencing from wood samples obtained throughout the USS *Cairo* ship structure.

## 2. Materials and Methods

**Figure 1:**
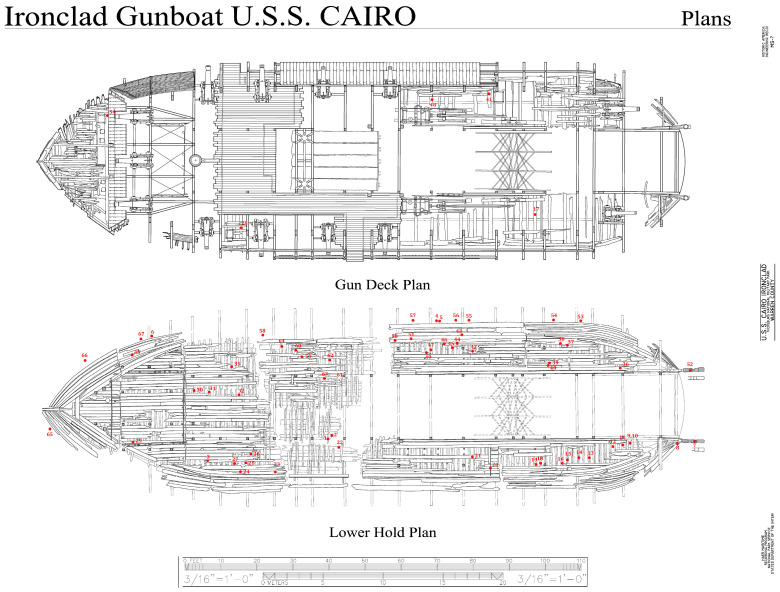
Diagram of the USS *Cairo* gunship, showing locations where wood samples were taken for analysis. A total of 67 samples, with red dots showing locations, were collected throughout the ship. Adapted from a photograph retrieved from the Library of Congress, <www.loc.gov/item/ms0291/> (accessed on 16 January 2025) Historic American Engineering Record, Creator, et al., photographed by Lowe, Jet, and Todd A Croteau. U.S.S. *Cairo* Ironclad, Vicksburg, Warren County, MS.

Sixty-six samples of small wood segments, approximately 1 × 2 × 2 to 2 × 2 × 8 cm, were obtained from the ship wood at various locations to obtain a good representation of different wood throughout the ship (Figure 1). In addition, one sample of straw and other dried plant material that may have been an animal nest was also sampled. The samples were placed in sterile bags and kept cool until they were brought to the laboratory. Sections from each sample were cut to remove the wood surface and used for identifying the type of wood and for isolation on culture media. No surface sterilization was carried out, but the outer surface of the wood sample was removed before isolation. For culturing, samples were placed in different types of media, including (i) malt extract agar (MEA) with antibiotics (15 g malt extract and 15 g agar in 1000 mL distilled water amended with 0.1 g/L streptomycin sulfate added after autoclaving) and (ii) a semi-selective media for Basidiomycota (15 g of malt extract, 15 g of agar, 2 g of yeast extract, 0.06 g of benomyl with 0.1 g of streptomycin sulfate, and 2 mL of lactic acid added after autoclaving). These culture media were used because of previous success in investigations conducted to obtain diverse fungal taxa from wood [20,21]. Antibiotics were used to prevent bacterial growth, and benomyl was used in one culture medium to inhibit fast-growing Ascomycota so that slower-growing Basidiomycota could be isolated. Plates were incubated at 22 °C, and once growth appeared, pure cultures were transferred to additional MEA plates. Isolates of pure cultures were then used for DNA extraction and sequencing. Cultures were stored in the University of Minnesota Forest Pathology culture collection in the Department of Plant Pathology.

A subset of the wood samples were used for elemental analyses using inductively coupled argon plasma optical emission spectrometry (ICP). Sound pine and oak, cut from modern boards, were used as controls. Al, B, Ba, Be, Ca, Cd, Co, Cr, Cu, Fe, K, Li, Mg, Mn, Mo, Na, Ni, P, Pb, Rb, Si, Sr, Ti, V and Zn were determined simultaneously by inductively coupled plasma atomic emission spectrometry (ICP-AES). A 500 mg sample of each type of wood was dried and ground and used in the analyses, with methods previously described by Held et al. [21]. Results are presented in ppm for each element in the samples.

Wood identification of each ship timber sample was conducted to identify the wood species using microscopic analysis of anatomical structures [22]. Additional wood sections were also prepared for scanning electron microscopy using previously described methods [23]. Observations were made, and images were taken using a Hitachi S3500 scanning electron microscope (Hitachi, Tokyo, Japan). The type of decay was determined by examination of the micromorphological characteristics of the decayed wood caused by different types of wood-destroying microorganisms [2,10,13,17].

The molecular identification of fungal cultures was completed by sequencing the internal transcribed spacer region of rDNA and comparing sequences to those in NCBI GenBank. DNA extraction, PCR conditions, sequencing, and sequence analysis followed the methods of Blanchette et al. [24]. Identification of cultures was based on the highest BLASTn algorithm using the megablast option in NCBI GenBank of a genus–species accession from a taxonomic study.

## 3. Results

**Figure 2:**
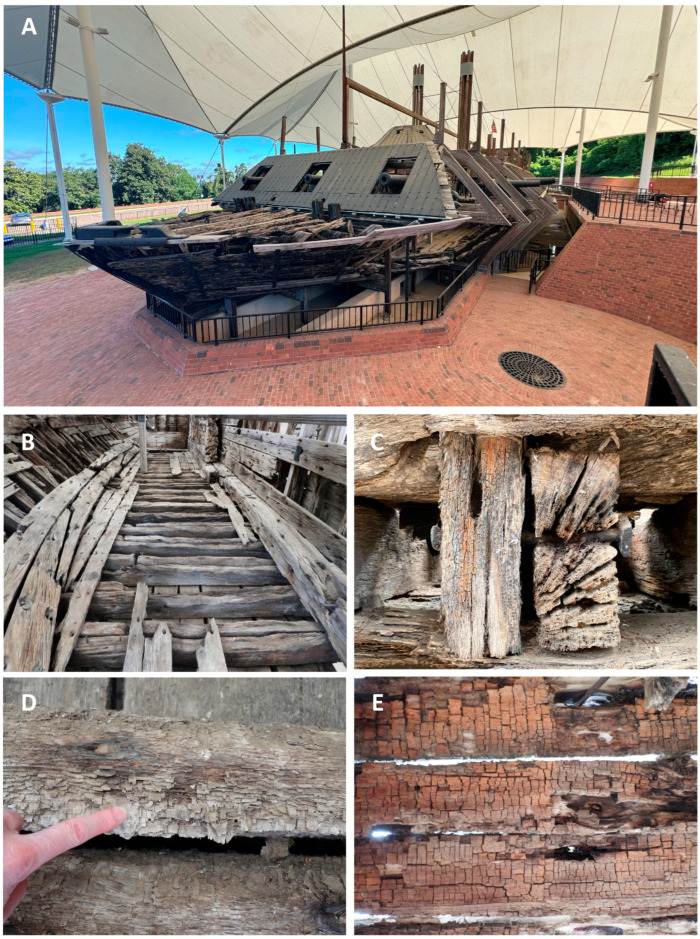
(**A**–**E**) The current condition of the ship and wood timbers. (**A**) The USS *Cairo* on display and under a protective canopy at the Vicksburg National Military Park in Vicksburg, Mississippi. (**B**,**C**) Ship timbers are eroded and degraded with large cracks evident. (**D**) White rot can be found in some woods where extensive degradation has resulted in most cells being destroyed except for the medullary ray parenchyma cells that resisted degradation. (**E**) Brown cubical rot typical of many brown rot fungi was found in some of the ship timbers.

**Figure 3:**
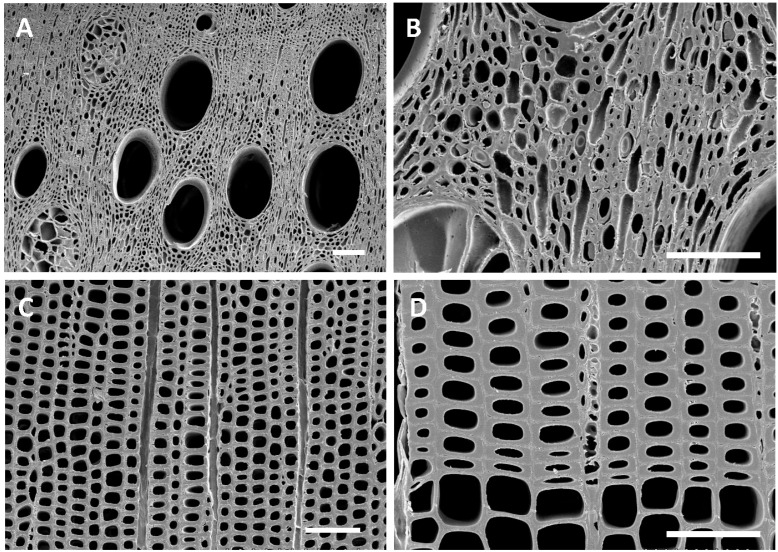
(**A**–**D**) Transverse sections of modern sound white oak (**A**,**B**) and pine (**C**,**D**) wood showing cells with intact and unaltered cell walls. Bar = 100 µm.

**Figure 4:**
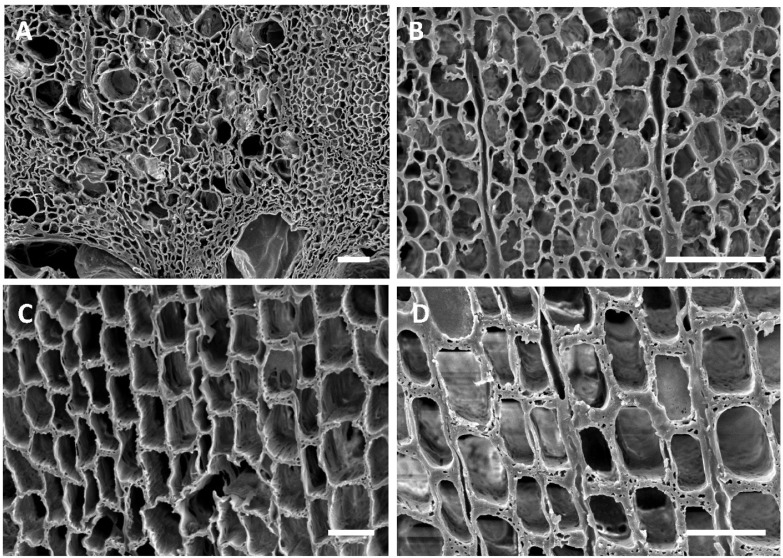
(**A**–**D**) Transverse sections of oak (**A**,**B**) and pine (**C**,**D**) wood from the USS *Cairo*. (**A**,**B**) The overall cell structure was evident in the wood but the secondary cell wall layers were degraded. Fiber cells had secondary walls that were eroded and thinned, leaving only the middle lamella. These thinned cell walls had characteristics of soft rot Type II attack. (**C**,**D**) Tracheids of pine wood sections showed distinct cavities withing the secondary walls, appearing as holes in the transverse sections, which were characteristic of Type I soft rot. Bar in (**A**,**B**,**D**) = 50 μm; (**C**) = 150 μm.

The ship is currently on display at the Vicksburg National Military Park, and various types of wood degradation are evident in the ship timbers (Figure 2). All timbers had noticeable degradation that was not easily characterized without microscopic observation (Figure 2). However, some wood exhibited distinct characteristics of white rot, where most of the wood cells were decayed, leaving the medullary ray parenchyma cells and brown rot with cubical checking of the wood (Figure 2). Anatomical observations of sections from the ship timbers indicated that most of the wood samples were oak (white oak group) with some pine and a few timbers that were yellow poplar (*Liriodendron tulipifera*) (Table S1). One sample of non-wood material was made up of straw and other fibrous plant material. Micromorphological characteristics of the wood observed with scanning electron microscopy showed that different types of degradation were present when compared to micrographs of sound modern white oak and pine wood. Sound wood had vessels, fibers, and parenchyma cells that were intact and unaltered (Figure 3). The sound pine had intact trachieds and parenchyma cells with no evidence of degradation (Figure 3). Sections of wood from all timbers examined showed different amounts of degradation. A common feature in the oak timbers was degradation of the secondary cell walls (Figure 4). Many fiber cells had completely degraded secondary walls with only the middle lamella remaining. The cell structure was still evident, but these thinned cells were greatly altered from those observed in sound wood and appeared with characteristics of Type II soft rot caused by fungi. In sections of pine wood, cavities were observed within secondary walls of trachieds that were characteristic of Type I soft rot attack (Figure 4).

**Figure 5:**
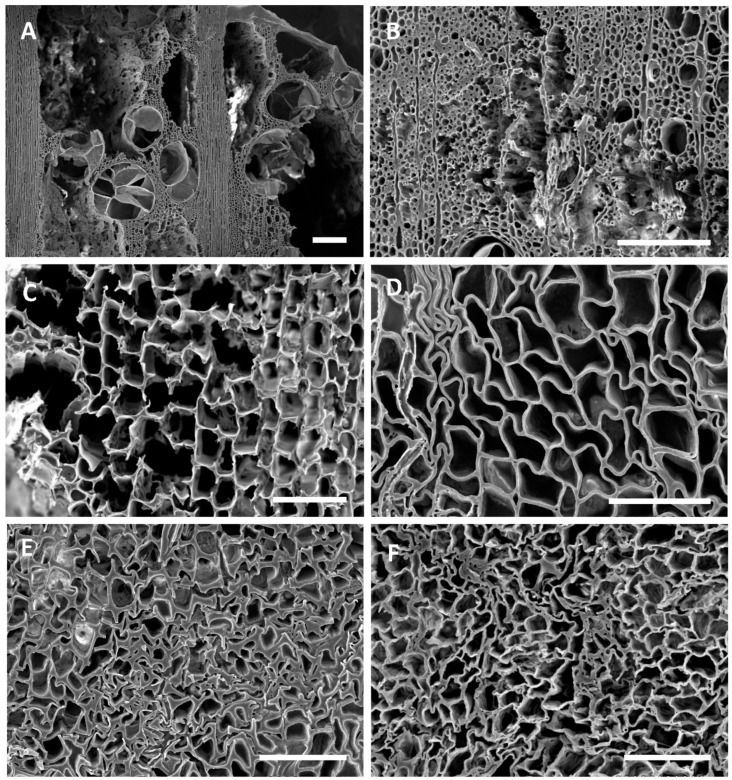
(**A**–**F**) Transverse sections of oak (**A**,**B**,**F**) and pine (**C**,**D**,**E**) showing various types of degradation. (**A**,**B**) Degradation was advanced in localized areas of many oak timbers, with all cells degraded, leaving voids within the wood. (**C**) Degradation typical of that caused by white rot fungi with erosion of all cell wall layers taking place. Both the secondary cell wall layers and middle lamella were degraded in some cells. (**D**) Degradation typical of brown rot fungi with cells that lack rigidity due to the loss of cellulose. Cells were distorted in shape and appeared convoluted. (**E**,**F**) Cells with degraded secondary walls were exceedingly weak and cells collapsed and were easily crushed. Bar in (**A**,**B**) = 200 μm; (**C**–**E**) = 100 μm; (**F**) = 50 μm.

Many of the timbers showed severe degradation. Sections from these wood samples displayed advanced stages of decay with many completely degraded cells (Figure 5). In these areas, all cell wall layers were degraded, including the middle lamella, leaving voids in the wood. Localized areas of disrupted and altered cells were also observed throughout many of the samples (Figure 5). Degradation with micromorphological characteristics of fungal white rot and brown rot (Figure 5) was evident in different timbers. With the white rot form of decay, secondary cell wall layers were eroded, including parts of the middle lamella between cells. The brown rot had cells with some structure remaining, but these cells had little strength left and the weak cell walls were convoluted and lacked rigidity. The oak and pine timbers from many areas of the ship were exceedingly fragile, and these areas showed eroded secondary wall layers with collapsed and crushed cells (Figure 5).

Culturing segments from the 67 samples taken from the ship followed by DNA extraction and sequencing revealed a diverse group of fungi (Table 1). Genera isolated most frequently in the Ascomycota included *Alternaria*, *Cladosporium*, *Curvularia*, *Epicoccum* and *Fusarium* and the Basidiomycota genera included *Bjerkandera*, *Coprinellus*, *Odontoefibula*, *Phanerodontia*, *Phlebiopsis*, *Roseograndinia* and *Trametes*. Many other taxa were also isolated in low frequency. Several samples from the timbers had more than one species isolated from it, and 19 of them had no viable fungi growing after culturing (Table S1). ITS sequences for the taxa are accessioned in GenBank, and accession numbers are listed in Table 1.

Elemental analyses using ICP of a subset of samples taken from throughout the ship showed higher levels of all elements tested as compared to those in sound oak and pine wood (Table 2). High amounts of boron were present in all samples, ranging from approximately 2000 to 50,000 mg/kg, with eight samples having over 10,000 mg/kg. Elevated levels of metals including aluminum, copper, iron, lead and zinc were also found, as well as high levels of calcium, magnesium, manganese, sodium and sulfur (Table 2).

## 4. Discussion

The USS *Cairo* has been subjected to many different environments since sinking in 1862. For over 98 years, it was in waterlogged conditions, with sediments accumulating that gradually buried the ship. Previous research on other sunken ships and waterlogged wood suggests that bacterial degradation and soft rot attack likely would have occurred during that extended period of time it was underwater [10,13,25,26]. The loss of wood strength observed in the USS *Cairo* when the ship was recovered from the bottom of the river also strongly suggests significant degradation had taken place [11]. Micromorphological observations showed that both oak and pine timbers had soft rot attack (Figure 4). Type I soft rot with cavities within the secondary cell wall layers of tracheids was prevalent in the pine wood, and Type II soft rot with erosion of the secondary wall was seen in the oak wood examined. Although clear evidence of erosion and tunneling bacterial degradation was not seen, bacterial attack undoubtedly took place and some of the eroded cell walls were likely caused by erosion bacteria while the ship was waterlogged. Since changes in the cell structure of the degraded waterlogged wood occurred after drying, much of the erosion that took place in the oak wood secondary cell walls could have been disrupted, and therefore they are not clearly identifiable as being caused by bacterial degradation. There were also areas in the timbers with advanced stages of decay and severe cell wall disruption (Figure 5). Many localized areas had completely degraded cells, and voids in the wood were present. In most cells, the thick secondary walls of the fibers were completely degraded, leaving just a framework of middle lamella remaining. This has resulted in an extremely fragile and weak wood cell wall structure. In these areas, cells often were found collapsed and crushed (Figure 5). This was due to the combined effects of bacterial and fungal attack along with physical stress caused by drying of the wood after recovery. The edges of the wood had more extensive degradation than interior parts of large timbers. There is also evidence of white and brown rot fungal attack in some wood (Figure 5). Since the ship was stored unsheltered outside for many years after it was recovered, there was ample opportunity for fungi to colonize and decay the wood. Identifying white and brown rot is possible since they have micromorphological signatures that can be differentiated using scanning electron microscopy [1,2,17]. It was reported that the ship wood was also intermittently sprayed with freshwater, intended to prevent deterioration, but unfortunately it produced a microenvironment conducive to rot [11]. Currently, the ship is protected under a tensile fabric canopy installed in 2000, and this open-sided structure protects against direct rainfall but not from the high temperatures and humidity common in Vicksburg, Mississippi.

A major question concerning the long-term preservation of the historic ship is the presence of viable wood-destroying fungi in its timbers that may be actively degrading the wood or could become more aggressive when conditions for their growth are optimum. Fungal isolation from the timbers and DNA sequencing revealed that a large diverse population of fungi are present in most timbers. These included many Ascomycota species that are known to cause soft rot, including *Alternaria*, *Curvularia*, *Chaetomium*, *Cladosporium*, *Xylaria* and others. Previous investigations have reported that these fungi can often be isolated from preservative-treated woods [27]. *Alternaria* was the most frequently isolated taxa and identified 12 times in different timbers. This fungus has been reported by many investigators to cause soft rot in conifers and hardwoods [28,29,30]. *Curvularia* has also been frequently isolated from preservative-treated wood and found to cause soft rot in pine as well as birch wood [27]. Other fungi isolated, such as *Chaetomium* and *Xylaria*, are also well known for their ability to produce soft rot [31,32,33]. In addition, several wood-destroying Basidiomycota were found, representing many different types of white rot fungi, including *Odontoefibula*, *Phanerodontia*, *Phlebiopsis*, *Roseograndinia*, *Trametes* and *Vitreoporus*. One of the more frequently isolated fungi was *Phanerodontia chrysosporium* (synonym is *Phanerochaete chrysosporium*). This fungus prefers higher temperatures than other wood decay fungi for growth, ranging from 30 to 37 °C, which is considered optimum for this species [34] but would not be for other white rot fungi. Many of these white rot fungi that were isolated from the ship are aggressive wood decay fungi that are commonly found in forests of Mississippi, but fungi such as *Odontoefibula orientalis* and *Roseograndinia minispora* have not been previously reported in North America. This may be due to their relatively obscure fruiting bodies, which are difficult to identify, and these species have only recently been named. Surprisingly, no brown rot fungi were isolated from any of the timbers, which may have been inhibited by the presence of boron in the wood.

Environmental conditions and substrates are important factors that dictate which microorganisms are established and grow in wood. For the USS *Cairo*, moisture in the wood from humidity at the site, temperature, compounds absorbed during burial from the river sediments, and elements such as boron and other preservatives that were applied to the ship after recovery would have had an influence on which fungi may colonize the wood. Elemental analyses indicated that the ship’s timbers had a very different elemental profile as compared to sound oak and pine wood. High concentrations of boron, copper, lead, zinc and many other metal ions were found in all of the wood sampled. Some wood had exceedingly high concentrations of up to approximately 50,000 ppm of boron and 700 ppm of copper, while others had less (Table 2). These elements likely originated from various past treatments with sodium borates and pentachlorophenol [11]. Other compounds such as calcium, iron and sulfur may have also accumulated from preservative treatments or increased in concentration while the ship was waterlogged and covered with sediments. Previous investigations on sunken ships in marine environments have demonstrated the accumulation of iron and sulfur in waterlogged wood [35,36]. In near-anaerobic environments, erosion bacteria degrade waterlogged woods, and many scavenging bacteria that utilize residual cellulose and other cell wall-degradative products reduce sulfate and promote the accumulation of low-valent sulfur compounds [15]. These ions can react with corroding iron objects in the ship, producing iron sulfides. This can also occur in freshwater systems but likely to a lesser extent than in marine environments [37].

The presence of many viable soft rot and white rot fungi isolated from the USS *Cairo* wood indicates that these species may tolerate metal ions and the boron that is present. It is known that some white rot fungi, including *Trametes versicolor*, can tolerate copper [38], and several studies have shown that some white rot fungi such as *Phanerodontia (Phanerochaete) chrysosporium* can degrade pentachlorophenol [39,40]. Many of the other white rot fungi isolated have not received much research attention and have not been tested for their ability to degrade wood preservatives. They would be good candidates to study since the results reported here indicate they are associated with wood with elevated concentrations of boron, and they appear to have tolerance to it. This ability to tolerate boron has not been previously investigated. The soft rot fungi that were isolated, such as *Alternaria*, *Curvularia*, *Chaetomium*, and *Cladosporium*, are taxa that have all been previously isolated from preservative-treated woods [27]. It is clear from the study we report here that these soft rot fungi can tolerate the various compounds used for preserving wood and remain alive in the wood. They could also be contributing to the biodegradation of these compounds as well; however, more work needs to be carried out to determine the extent and rate of degradation

No brown rot fungi were isolated from any of the samples cultured, but there was evidence of brown rot decay in a few wood samples (Figure 1) when observed with scanning electron microscopy (Figure 5). Some of these areas had rot that was dark brown, brittle, and fractured into cubical pieces, which is typical for brown-rotted wood (Figure 2). This brown rot attack may have taken place after recovery of the ship and while it was unsheltered for over ten years. The various preservative applications were likely effective against the brown rot fungi since there were no viable brown rot fungi isolated. However, other fungi, including white and soft rot fungi, were present. Although they were alive and readily isolated from the wood, their extent of colonization and rate of wood degradation are not known. These fungi could also be in an inactive state within the wood or could reactivate, causing new degradation, whenever conditions are favorable for growth. The large number of diverse fungal taxa that are present in the ship’s wood raises concerns about the future preservation of the ship. Additional concerns include the fluctuation in temperature and humidity, which may affect the fragile residual cells of the ship wood. Dust and debris entering the ship, as well as the presence of insects, birds and other animals that may cause damage to the ship in its current open-sided canopy, are also a concern. A critical factor for controlling fungal degradation of wood is to remove moisture. For the USS *Cairo*, this can best be accomplished by constructing a building for the ship with environmental control. Keeping relative humidity below 55% would arrest any fungal degradative actions. An enclosed structure would also prevent dust, insects and animals from interacting with the ship. Undoubtedly, the condition of the wood will continue to deteriorate if the existing biodeterioration and biodegradation processes underway in the ship are left unaddressed.

This investigation provides important new information that will aid in conservation efforts to preserve the historic USS *Cairo*. The micromorphological results provide a better understanding of the current condition of the wood and help to unravel past decomposition processes that have occurred as the ship has been subjected to different environmental conditions. The fungal isolation results and presence of so many fungi with the capacity to degrade wood also suggest that there is a need for additional studies to better understand how soft rot and white rot fungi tolerate and interact with aging wood that has been previously treated with wood preservation compounds.

## Figures and Tables

**Table 1:** List of taxa and frequency of isolation from 67 timber samples obtained from the USS *Cairo* with GenBank accession numbers.

Fungal Taxa	Occurrence Count	GenBank #
*Agaricomycetes* sp.	1	PV664712
*Alfaria* sp.	1	PV664710
*Alternaria* sp.	12	PV664711
*Arthrinium* sp.	1	PV664713
*Athelia* sp.	1	PV664714
*Bjerkandera adusta*	4	PV664715
*Ceratostomella crypta*	1	PV664716
*Chaetomium* sp.	2	PV664717
*Cladosporium dominicanum*	7	PV664718
*Cladosporium halotolerans*	1	PV664719
*Cladosporium malorum*	2	PV664720
*Cladosporium* sp.	7	PV664721
*Cladosporium sphaerospermum*	2	PV664722
*Coprinellus aureogranulatus*	1	PV664723
*Coprinellus disseminatus*	1	PV664724
*Coprinellus radians*	1	PV664725
*Curvularia intermedia*	1	PV664726
*Curvularia* sp. 1	3	PV664727
*Curvularia* sp. 2	11	PV664728
*Curvularia* sp. 3	4	PV664729
*Curvularia* sp. 4	2	PV664730
*Curvularia* sp. 5	1	PV664731
*Elmerina phellinoides*	1	PV664732
*Epicoccum nigrum*	1	PV664733
*Epicoccum* sp.	3	PV664734
*Exserohilum rostratum*	1	PV664735
*Fusarium* sp.	1	PV664736
*Fusarium* sp. 1	3	PV664737
*Fusarium* sp. 2	1	PV664738
*Leptosphaerulina* sp.	1	PV664739
*Mucronella* sp.	1	PV664740
*Myrmecridium schulzeri*	1	PV664741
*Nemania* sp.	1	PV664742
*Neopestalotiopsis* sp.	2	PV664743
*Neurospora* sp.	1	PV664744
*Nigrospora sphaerica*	1	PV664745
*Nothophoma quercina*	1	PV664746
*Odontoefibula orientalis*	1	PV664747
*Paraphaeosphaeria burbidgeae*	1	PV664748
*Penicillium* sp. 1	1	PV664749
*Penicillium* sp. 2	2	PV664750
*Periconia epilithographicola*	2	PV664751
*Peroneutypa scoparia*	2	PV664752
*Pestalotiopsis* sp.	2	PV664753
*Phaeosphaeriopsis musae*	1	PV664754
*Phanerodontia chrysosporium*	6	PV664755
*Phanerodontia magnoliae*	1	PV664756
*Phlebiopsis flavidoalba*	2	PV664757
*Plectosphaerella cucumerina*	1	PV664758
*Roseograndinia minispora*	1	PV664759
*Trametes versicolor*	3	PV664760
*Vitreoporus dichrous*	1	PV664761
*Xylaria* cf. *heliscus*	1	PV664762

**Table 2:** Elemental analysis (ppm) of wood collected from various timbers in the USS *Cairo* and control sound wood used for comparison. Values below detectable limits are indicated with “-”.

Sample ID	Al	As	B	Ba	Ca	Cu	Fe	K	Mg	Mn	Na	P	Pb	S	Si	Sr	Ti	Zn
Control pine	6	-	2	3	630	1	14	421	216	19	-	37	-	43	13	3	-	7
Control Oak	2	-	5	8	327	2	9	477	29	7	-	41	-	42	8	1	-	1
Cairo #10	438	1	10,254	37	9291	114	1201	465	728	72	14,047	174	20	845	872	53	9	991
Cairo #13	620	1	9639	62	10,955	85	4687	782	1047	127	16,964	363	112	889	1505	63	13	903
Cairo #15	476	1	4463	33	6300	318	3448	839	643	76	12,278	270	75	2188	944	47	10	212
Cairo #19	2482	4	2248	79	15,132	180	15,154	889	1215	435	10,621	856	167	2353	3516	183	54	373
Cairo #20	1498	3	9258	64	11,613	212	6302	840	1288	390	14,187	546	707	2329	1714	93	26	923
Cairo #22	262	22	5352	89	13,156	201	11,777	1077	934	101	12,087	203	670	1540	564	124	8	652
Cairo #23	354	1	3917	32	10,625	22	8241	471	530	41	9985	165	8	571	587	42	8	96
Cairo #25	357	20	8342	32	4361	106	5961	226	467	91	7820	218	51	378	448	33	6	367
Cairo #26	2763	200	5468	83	11,073	287	9631	608	1160	417	9252	683	225	1493	2342	69	32	941
Cairo #29	349	2	1974	57	11,326	522	9440	577	696	141	7421	306	56	3726	560	111	8	692
Cairo # 33	276	4	18,412	40	9400	735	10,541	825	675	118	23,448	308	166	4528	385	102	6	563
Cairo #36	466	1	5763	36	6075	28	1222	659	552	53	9474	169	141	736	671	33	11	224
Cairo #40	327	1	20,667	31	5517	175	1777	490	647	75	31,802	163	144	1778	642	49	5	244
Cairo #41	756	1	20,445	29	6532	85	1144	765	792	70	32,025	280	22	1390	1118	29	12	391
Cairo #48	1034	3	49,940	67	10,619	160	4275	868	992	272	52,067	568	279	1529	1244	62	22	814
Cairo #50	249	1	20,991	52	3896	76	5793	484	440	54	21,749	211	49	902	355	29	5	81
Cairo #19	910	2	18,165	54	8766	205	8467	1062	793	130	42,630	402	131	4027	1688	63	16	260
Cairo #60	894	2	6209	53	11,886	714	8380	1017	873	89	32,236	373	47	4811	1725	86	17	125
Cairo #64	526	33	20,517	97	10,487	237	33,022	1013	810	81	35,802	300	358	2031	1770	124	12	1871

## Data Availability

The original contributions presented in this study are included in the article/Supplementary Materials. Further inquiries can be directed to the corresponding author.

## Supplementary Materials

The following supporting information can be downloaded at https://www.mdpi.com/article/10.3390/jof11100732/s1. Table S1: List of taxa isolated from each timber sample with % identity to known fungal sequences in GenBank. Identification was based on the highest BLAST match score of a genus–species accession from a taxonomic study.
