# Supplementary material for: Evaluation of Wood Decay and Identification of Fungi Found in the USS Cairo, a Historic American Civil War Ironclad Gunboat
Source: J Fungi (Basel). 2025 Oct 11;11(10):732. doi: 10.3390/jof11100732 (PMC12565250; doi:10.3390/jof11100732)
Supplement: Supplementary file 1 [file jof-11-00732-s001.zip › jof-3874192-supplementary.pdf]

Supplemental Table S1. List of wood identification and taxa isolated and identified from each timber sample with % identity and % sequence coverage to known fungal sequences in GenBank. Identification was based on the highest BLAST match score of a genus-species accession from a taxonomic study.

Cairo Sample Number	Fungal Taxa	Query Coverage	Max Identity	Sample Wood ID
Cairo-1	*Cladosporium dominicanum*	100%	99%	*Quercus* sp.
Cairo-2	*Alfaria* sp.	99%	99%	*Quercus* sp.
	Cladosporium malorum	100%	99%	
	Curvularia sp. 2	100%	99%	
	Curvularia sp. 2	100%	100%	
	Curvularia sp. 4	100%	100%	
	Elmerina phellinoides	100%	99%	
	Fusarium sp.	100%	99%	
	Fusarium sp. 1	100%	100%	
	Phanerodontia chrysosporium	100%	100%	
Cairo-3	*Coprinellus aureogranulatus*	100%	99%	*Quercus* sp.
	Curvularia sp. 1	100%	99%	
	Curvularia sp. 3	100%	99%	
	Phanerodontia chrysosporium	100%	99%	
Cairo-4	*Cladosporium dominicanum*	99%	99%	*Quercus* sp.
Cairo-5	--	--	--	*Quercus* sp.
Cairo-6	*Curvularia* sp. 2	100%	100%	*Quercus* sp.
	Penicillum sp. 1	100%	100%	
	Phanerodontia chrysosporium	100%	99%	
Cairo-7	*Curvularia* sp. 2	100%	99%	*Lirodendron tulipifera*
	Mucronella sp.	99%	97%	
	Myrmecridium schulzeri	100%	99%	
Cairo-8	*Phanerodontia chrysosporium*	100%	100%	*Quercus* sp.
Cairo-9	*Paraphaeosphaeria burbidgeae*	100%	100%	*Quercus* sp.
Cairo-9	*Periconia epilithographicola*	100%	100%	*Quercus* sp.
Cairo-10	--	--	--	*Quercus* sp.
Cairo-11	--	--	--	*Quercus* sp.
Cairo-12	--	--	--	*Quercus* sp.
Cairo-13	--	--	--	*Quercus* sp.
Cairo-14	*Bjerkandera adusta*	99%	99%	*Quercus* sp.
	Cladosporium dominicanum	100%	99%	
Cairo-15	--	--	--	*Quercus sp.*
Cairo-16	*Bjerkandera adusta*	100%	100%	*Quercus* sp.
Cairo-17	--	--	--	*Quercus* sp.
Cairo-18	--	--	--	*Quercus* sp.
Cairo-19	*Alternaria* sp.	100%	100%	*Quercus* sp.
	Fusarium sp. 1	100%	99%	
Cairo-20	*Alternaria* sp.	100%	100%	*Pinus* sp.
	Athelia sp.	99%	99%	
	Bjerkandera adusta	100%	99%	
	Cladosporium sp.	100%	99%	
	Roseograndinia minispora	100%	98%	
Cairo-21	--	--	--	*Pinus* sp.
Cairo-22	--	--	--	*Quercus* sp.

Cairo-23	*Agaricomycetes* sp.	100%	99%	*Quercus* sp.
	Cladosporium sp.	100%	100%	
	Pestalotiopsis sp.	100%	100%	
Cairo-24	*Alternaria* sp.	100%	100%	*Quercus* sp.
	Chaetomium sp.	100%	100%	
	Epicoccum sp.	100%	100%	
Cairo-25	*Ceratostomella crypta*	100%	99%	*Quercus* sp.
	Cladosporium sp.	100%	100%	
	Coprinellus radians	100%	100%	
	Neopestalotiopsis sp.	100%	100%	
	Neurospora sp.	100%	99%	
	Odontoefibula orientalis	100%	100%	
	Peroneutypa scoparia	100%	97%	
Cairo-26	*Epicoccum nigrum*	100%	99%	*Pinus* sp.
	Fusarium sp. 1	100%	100%	
	Phanerodontia chrysosporium	100%	100%	
Cairo-27	--	--	--	*Quercus* sp.
Cairo-28	*Cladosporium* sp.	100%	100%	*Pinus* sp.
	Curvularia sp. 2	100%	100%	
	Curvularia sp. 3	100%	100%	
Cairo-29	--	--	--	*Quercus* sp.
Cairo-30	*Vitreoporus dichrous*	100%	100%	*Quercus* sp.
Cairo-31	--	--	--	*Quercus* sp.
Cairo-32	*Neopestalotiopsis* sp.	100%	100%	*Quercus* sp.
Cairo-33	*Nigrospora sphaerica*	100%	100%	*Quercus* sp.
Cairo-34	--	--	--	*Quercus* sp.
Cairo-35	*Curvularia* sp. 2	100%	100%	*Quercus* sp.
	Epicoccum sp.	100%	99%	
Cairo-36	*Alternaria* sp.	100%	100%	*Pinus* sp.
	Fusarium sp. 2	100%	100%	
	Trametes versicolor	100%	100%	
Cairo-37	*Cladosporium dominicanum*	100%	99%	*Pinus* sp.
	Curvularia sp. 3	100%	100%	
Cairo-38	*Alternaria* sp.	100%	100%	*Pinus* sp.
	Cladosporium sp.	100%	100%	
	Curvularia sp. 2	100%	100%	
	Nemania sp.	100%	100%	
	Penicillium sp. 2	100%	100%	
	Phlebiopsis flavidoalba	100%	100%	
	Phlebiopsis flavidoalba	100%	100%	
Cairo-39	*Bjerkandera adusta*	100%	99%	Straw, branches
Cairo-40	*Alternaria* sp.	100%	99%	*Pinus* sp.
Cairo-41	*Cladosporium* sp.	100%	100%	*Pinus* sp.
	Curvularia sp. 2	100%	100%	
	Trametes versicolor	100%	100%	
Cairo-42	*Alternaria* sp.	100%	99%	*Quercus* sp.
	Cladosporium sp.	100%	100%	
Cairo-43	*Penicillium* sp. 2	100%	100%	*Quercus* sp.
Cairo-44	*Periconia epilithographicola*	100%	100%	*Quercus* sp.
Cairo-45	*Phanerodontia magnoliae*	100%	100%	*Quercus* sp.
	Trametes versicolor	100%	100%	
Cairo-46	*Alternaria* sp.	100%	100%	*Quercus* sp.
Cairo-47	*Peroneutypa scoparia*	100%	100%	*Quercus* sp.

Cairo-48	*Alternaria* sp.	100%	100%	*Pinus* sp.
	Cladosporium dominicanum	100%	100%	
	Curvularia sp. 5	100%	99%	
Cairo-49	--	--	--	*Quercus* sp.
Cairo-50	*Chaetomium* sp.	100%	100%	*Quercus* sp.
	Curvularia sp. 2	100%	100%	
Cairo-51	--	--	--	*Quercus* sp.
Cairo-52	*Cladosporium sphaerospermum*	100%	99%	*Lirodendron tulipifera*
	Coprinellus disseminatus	100%	100%	
	Curvularia sp. 2	100%	100%	
	Phaeosphaeriopsis musae	100%	100%	
Cairo-53	--	--	--	*Quercus* sp.
Cairo-54	--	--	--	*Quercus* sp.
Cairo-55	*Curvularia intermedia*	100%	100%	*Quercus* sp.
	Curvularia sp. 2	100%	100%	
	Exserohilum rostratum	100%	100%	
	Nothophoma quercina	100%	99%	
Cairo-56	*Cladosporium halotolerans*	100%	100%	*Quercus* sp.
Cairo-57	*Alternaria* sp.	100%	100%	*Pinus* sp.
	Curvularia sp. 4	100%	100%	
	Epicoccum sp.	100%	100%	
	Plectosphaerella cucumerina	100%	99%	
Cairo-58	*Alternaria* sp.	100%	100%	*Quercus* sp.
	Pestalotiopsis sp.	100%	100%	
Cairo-59	*Curvularia* sp. 1	100%	98%	*Quercus* sp.
Cairo-60	*Xylaria cf. heliscus*	100%	100%	*Quercus* sp.
Cairo-61	*Leptosphaerulina* sp.	100%	100%	*Quercus* sp.
	Phanerodontia chrysosporium	100%	100%	
Cairo-62	*Cladosporium dominicanum*	100%	100%	*Quercus* sp.
Cairo-63	*Arthrinium* sp.	100%	99%	*Quercus* sp.
	Curvularia sp. 3	100%	100%	
Cairo-64	--	--	--	*Quercus* sp.
Cairo-65	*Cladosporium sphaerospermum*	100%	100%	*Pinus* sp.
	Curvularia sp. 2	100%	100%	
Cairo-66	*Cladosporium dominicanum*	100%	100%	*Quercus* sp.
	Curvularia sp. 1	100%	100%	
Cairo-67	*Alternaria* sp.	100%	100%	*Pinus* sp.
	Cladosporium malorum	100%	100%	

[a] Columns with "- -" indicate no fungi were isolated from the wood sample.
